# Greed and Fear in Network Reciprocity: Implications for Cooperation among Organizations

**DOI:** 10.1371/journal.pone.0147264

**Published:** 2016-02-10

**Authors:** James A. Kitts, Diego F. Leal, Will Felps, Thomas M. Jones, Shawn L. Berman

**Affiliations:** 1 Department of Sociology, University of Massachusetts, Amherst, Massachusetts, United States of America; 2 Australian School of Business, The University of New South Wales, Sydney, New South Wales, Australia; 3 Foster School of Business, University of Washington, Seattle, Washington, United States of America; 4 Anderson School of Management, University of New Mexico, Albuquerque, New Mexico, United States of America; Universidad Carlos III de Madrid, SPAIN

## Abstract

Extensive interdisciplinary literatures have built on the seminal spatial dilemmas model, which depicts the evolution of cooperation on regular lattices, with strategies propagating locally by relative fitness. In this model agents may cooperate with neighbors, paying an individual cost to enhance their collective welfare, or they may exploit cooperative neighbors and diminish collective welfare. Recent research has extended the model in numerous ways, incorporating behavioral noise, implementing other network topologies or adaptive networks, and employing alternative dynamics of replication. Although the underlying dilemma arises from two distinct dimensions—the gains for exploiting cooperative partners (Greed) and the cost of cooperating with exploitative partners (Fear)–most work following from the spatial dilemmas model has argued or assumed that the dilemma can be represented with a single parameter: This research has typically examined Greed or Fear in isolation, or a composite such as the K-index of Cooperation or the ratio of the benefit to cost of cooperation. We challenge this claim on theoretical grounds—showing that embedding interaction in networks generally leads Greed and Fear to have divergent, interactive, and highly nonlinear effects on cooperation at the macro level, even when individuals respond identically to Greed and Fear. Using computational experiments, we characterize both dynamic local behavior and long run outcomes across regions of this space. We also simulate interventions to investigate changes of Greed and Fear over time, showing how model behavior changes asymmetrically as boundaries in payoff space are crossed, leading some interventions to have irreversible effects on cooperation. We then replicate our experiments on inter-organizational network data derived from links through shared directors among 2,400 large US corporations, thus demonstrating our findings for Greed and Fear on a naturally-occurring network. In closing, we discuss implications of our main findings regarding Greed and Fear for the problem of cooperation on inter-organizational networks.

## Introduction

In this paper, we investigate a classic model of the evolution of cooperation—the spatial dilemmas model of Nowak and May [1, 2]–and apply it to the problem of cooperation among organizations. The model considers social dilemmas, where agents may be driven to behave in non-cooperative ways either to exploit others’ generosity (Greed) or to avoid exploitation by others (Fear). Although classical game theory assumes that variations in Greed and Fear have no effect within the Prisoner’s Dilemma, and work on the spatial dilemmas model typically assumes that Greed and Fear have similar effects, we demonstrate that Greed and Fear have strong, divergent, and interactive effects in the model. Implementing ‘virtual interventions’ to Greed and Fear, we show that some such changes also have temporally asymmetric effects: Due to changes in the diffusion potential of strategies at critical values of Greed and Fear, interventions to decrease Fear or increase Greed may lead to effects on cooperation that maintain even after the intervention is fully reversed. Stepping beyond the spatial dilemmas interaction topology, we apply the model to a network of empirical inter-organizational relationships and demonstrate robustness of our conclusions for a realistic network topology. In closing, we discuss the distinct implications of Greed and Fear for organizational contexts.

The spatial dilemmas model builds on the Prisoner’s Dilemma (PD) describing the strategic interdependence of two parties: If both parties *cooperate*, each will receive a payoff represented by the symbol CC (for a Cooperator’s payoff with another Cooperator). If both *defect*, then each will receive a lower payoff (DD: a Defector’s payoff with another Defector). If only one of the partners defects, that actor receives a payoff (DC) greater than that offered by mutual cooperation while the lone cooperator receives a payoff (CD) lower than that offered by mutual defection. The PD formally requires that:

1 DC > CC > DD > CD
2 2CC > DC + CD (Mutual cooperation is more collectively productive than exploitation.)

For the substantive contexts that we consider, it also makes sense to require that:

3 DC + CD > 2DD (Cooperation of one player is more collectively productive than the cooperation of neither player.)

The dilemma in the payoffs above is that the optimal strategy for each *individual* player is defection, which leads to a suboptimal *collective* outcome.

In the classic (1-play; 2-player) PD, defectors (D) will always outperform cooperators (C). However, C performs better when interacting with other Cs than D performs when interacting with other Ds; that is, CC > DD. Therefore, once more than two players are admitted, the relative performance of each strategy depends on the distribution of other strategies in the environment. It is well established that in a population where interaction is unstructured (mixing Ds with Cs arbitrarily), unconditional cooperation will be doomed, though cooperation can survive if Cs interact primarily with other Cs and Ds interact primarily with other Ds [3]. That is, certain structures of interaction may make cooperation pay.

In the spatial dilemmas model and work following from it, interactions are embedded in networks rather than occurring arbitrarily in the entire population. This assumption of embeddedness mirrors the empirical reality that social dilemmas often occur not among randomly sorted players but among clusters of peers [4]. The model employs the Moore neighborhood, one of the simplest and well-studied topologies: a regular lattice where each agent interacts with its eight immediate neighbors (N, E, S, W, NE, SE, SW, and NW). The matrix ‘wraps’ back on itself, matching the top with the bottom and the left side with the right side (forming a torus) so that there are no edges or corners and thus no central or peripheral agents. Importantly, this prototypical network assumes a moderate level of local clustering in network ties, where some neighbors are tied to their neighbors’ neighbors.

A population of agents is distributed randomly across this lattice. Each agent plays simultaneously in PD games with all neighbors in each round. After cooperating with (or defecting on) all eight of its neighbors, each agent receives a payoff from each game and its performance is the sum across payoffs. Agents are aware of their own performance and the performance of immediate neighbors at the end of each round. If an agent is outperformed by at least one neighbor, it imitates its most successful neighbor on the following round (or chooses randomly among the strategies of two or more neighbors who tie for highest performer). If no neighbors beat the focal agent, it keeps its current strategy. Subsequent rounds follow the same pattern; individual agents switch between cooperate and defect as the relative performance of the two strategies in their neighborhood changes. The general dynamic of replication according to relative fitness, a standard assumption in evolutionary game theory [5, 6], could formally represent a variety of empirical mechanisms—e.g., social learning, operant learning, imitation, or differential patterns of founding and failure. We describe this simple diffusion on networks as *social learning* [7].

Although the original model has been tremendously influential, numerous studies over the past two decades have criticized and extended it. First, the dynamics of the basic model are deterministic with synchronous updates of agents’ states, and some extensions have employed randomly asynchronous updates [8–12] or incorporated noise or mutation [11, 13, 14]. Second, researchers have questioned whether the two-dimensional grid is an adequate description of real world interpersonal and biological networks, and have extended the model to investigate more complex network structures [15] such as regular random graphs [15–18], small-world networks [16, 19, 20], or scale-free networks [18, 21–23]. Indeed, related studies have considered dynamic models of interaction for networks of binary ties (e.g., [24–27]), ties that varied in strength over time (e.g., [28–30]), and ties that also change positive or negative valence over time [31]. Third, recent work has questioned whether Nowak and May’s rule for local replication—that is, imitation of the fittest neighbor—is an accurate description of the diffusion of strategies. This doubt has been fueled by recent debates [32–36] on whether human subjects are indeed influenced by their neighbors in this way.

Given that the spatial dilemmas model has been taken to apply to practically any social dilemma among agents situated in social or biological networks, a central concern is whether the model’s dynamics are sufficiently general and robust to account for this vast domain of phenomena. If conclusions derived from the model depend on details such as the network topology or updating rule, then one approach is to continue the search for a more satisfactory general model to account for cooperation on networks across all contexts. A more tractable approach, which we use here, is to reduce the scope conditions to a particular domain where the challenged aspects of the model above are less problematic. For instance, in this paper we will apply the spatial dilemmas model to the problem of cooperation in inter-organizational networks.

While acknowledging limitations of the basic model, we will discuss how assumptions of the model may fit the empirical domain of inter-organizational cooperation. We will also criticize the classic model, but we will focus our critique on a core claim that Nowak and May made initially and that has been maintained through two decades of following research: Although the Prisoner’s Dilemma stems from two perverse incentives: the expected gain for actors who exploit cooperative partners (‘Greed’) and the expected loss for cooperative parties who are exploited by partners (‘Fear’), the vast majority of theoretical work on networks and social dilemmas has represented the PD as a *single* dimension, taken to represent the severity of the dilemma and claimed to represent the full range of the model’s behavior. Unlike this prior research, we distinguish Greed from Fear as distinct dimensions of the dilemma and manipulate them orthogonally in a fine-grained experiment. This allows us to see any interactions between Greed and Fear, and produce more nuanced insights into the emergence and stability of cooperation. For example, we identify conditions in which even modest changes in Greed and/or Fear have profound implications for cooperation; in other conditions, even large changes of Greed and/or Fear have little effect. We explore the model, showing that Greed and Fear have surprisingly divergent effects in the population, even without assuming that individual agents respond differently to Greed and Fear. We show that these macro level differences result from the embeddedness of interaction in networks.

We begin by (1) elaborating on two dimensions of social dilemmas (Greed and Fear). Next, we (2) present a computational experiment in which we manipulate dimensions of the spatial dilemmas model (Greed, Fear, and the initial level of cooperation). After presenting our main results, we (3) implement a set of ‘virtual interventions’ that change Greed or Fear over time, revealing how some such changes have impacts that are difficult to reverse, and again showing differences between Greed and Fear. We then (4) explore the adequacy of the Moore neighborhood in the spatial dilemmas model as a representation of inter-organizational networks, simulate the model on an empirical network of inter-organizational relationships, and discuss robustness of our conclusions to other specific assumptions of the model. Finally, we (5) discuss how efforts to remedy Greed and Fear are distinctly reflected in empirical policy interventions and identify some implications of our findings for informing those interventions.

### Two Ways to Promote Cooperation in Social Dilemmas: Reducing Greed or Fear

Extensive research has demonstrated that interventions to alter the payoffs of strategic choices may be a credible means of fostering cooperation in social dilemmas [37–39]. Within this domain, a more specific line of research contrasts two distinct problems underlying the PD, decomposing the game into three parameters representing the differences between the absolute payoffs in the game [40]. Using the previously defined payoff symbols, ‘Greed’ is the incremental benefit of exploiting a partner who unilaterally cooperates versus cooperating with that partner (DC—CC). ‘Fear’ is the incremental loss due to unilaterally cooperating versus mutual defection (DD—CD). Finally, ‘Value of exchange’ is the individual’s benefit of mutual cooperation versus mutual defection (CC—DD). These three differences between adjacent payoffs concisely represent the core definition of the PD in inequality (1), and we use the terms *Greed* and *Fear* exclusively to refer to these payoff differences in the game theoretic definition. (We do not refer to any cognitive or affective process that may be implied by common usage of the English words ‘Greed’ and ‘Fear’, which have concerned other scholars [41, 42].) By definition, Greed (G), Fear (F), and the Value (V) of exchange are all positive in the PD, where Greed and Fear are the two sources of the dilemma. Rapoport’s well-known ‘K-index of cooperation’ [40]–or (CC-DD)/(DC-CD)–can be represented as V/(F+G+V), which treats Greed and Fear as interchangeable and approaches its lower limit (0.0) in the PD when Greed or Fear are arbitrarily large and approaches its upper limit (1.0) when Greed and Fear are near zero.

Classical game theory focuses on the ordering of payoffs, assuming that the magnitudes of Greed and Fear make no difference within the PD, while previous research on spatial dilemmas treated Greed and Fear as interchangeable. Nevertheless, social psychologists have argued that we should distinguish Fear from Greed as dimensions of social dilemmas because humans may respond differently to these incentives. Much research on Greed and Fear (e.g., [8, 18, 43]) has explored a space of what we call the CD and DC payoffs, comparing the PD with other games: Stag Hunt (without Fear), Snowdrift (without Greed), and Harmony games (with neither Greed nor Fear). Behavioral research has produced mixed effects and has not given a clear answer as to when Greed or Fear will be a more important obstacle to cooperation among individuals. For example, several studies have found that people may be more sensitive to Greed than to Fear; increasing Greed diminishes the rate of cooperation in 2-player and multiplayer games, whereas increasing Fear has inconsistent or weaker effects [44–48]. However, other research finds that either Greed or Fear may undermine cooperation [49–52]. Given our interest in cooperation among organizations (whose strategies are developed by boards and management teams), it is not clear that micro-level personality traits, moods, or evolved heuristics that drive human behavior will translate into similar effects on organizational populations. Thus, we do *not* assume that corporate actors have different responses to Greed and Fear. Thus, any of our discoveries that Greed and Fear have distinct effects at the population level are interpretable as emergent properties of our evolutionary model, rather than reflecting a built-in assumption of differences in individuals’ responses to Greed and Fear. Also, unlike work comparing the PD to Stag Hunt, Snow Drift, or Harmony games, we focus exclusively on the PD, implementing small manipulations to Greed and Fear that do not alter the order of payoffs in inequality (1) but allow us to explore the fine-grained shape of the model’s behavior within the PD game.

Research on network reciprocity following from the spatial dilemmas model has generally focused on a simplified representation of the PD game that reduces the severity of the dilemma to one parameter. For example, many have manipulated Greed in isolation while setting Fear to zero [1, 21, 53–56] or a fixed positive value [24], while others have manipulated Fear in isolation while setting Greed to zero [57, 58]. Others still have set Fear = Greed and manipulated both jointly as ‘intensity’ of the dilemma [22, 59], or have employed composite measures, such as the ratio of the benefit to cost of cooperation [15, 60] or the ‘K-index’ of cooperation [40, 61]. In a fine-grained computational experiment, we will map the shape of cooperation over the space of Greed and Fear. In doing so, we will show that embedding social dilemmas in social space (such as locally clustered interaction networks) implies very different effects for Greed and Fear, and that these differences are substantial and robust. We will also show that temporal changes in Greed and Fear have very different implications.

### Strategy Replication Among Organizations: Mimetic Isomorphism

The idea of imitating higher performers in a network is the focus of a large body of theory (for an accessible introduction, see Skyrms [58] or Nowak and Sigmund [62]), but reviewing this work is outside the scope of our paper. Recent criticism of the classic spatial dilemmas model for assuming that agents imitate their fittest neighbor [9, 12, 22] has led to debates about whether this assumption indeed fits human behavior on networks [32–36]. Whether or not this assumption fits the behavior of individual humans, there is a considerable body of evidence that organizations imitate successful and proximate organizations through a combination of benchmarking performance against peers and migration of personnel between organizations [63–66]. Not only is the adoption of perceived ‘best practices’ a widely observed phenomenon in organization science, but managers often acknowledge doing this, and they are even expected to do so as part of their fiduciary duty to stockholders or other stakeholders. Institutional theorists call this conformity to perceived best practices among peers ‘mimetic isomorphism’ [67].

## Methods

### Experimental Design

In this experiment, our dependent variable is the average long-run prevalence of cooperation (proportion of agents cooperating). By “long-run” we mean the stable value of cooperation that the model approaches after an arbitrarily large number of rounds of interaction. The model is always allowed to iterate until it reaches this long-run behavior. In some cases, the model never reaches a micro-level stable equilibrium (i.e., where no single agent would ever change from C to D or D to C), but the simulation orbits narrowly around a system-level dynamic equilibrium. For example, one or more agents sitting on a boundary may endlessly flip back and forth between C and D, while the overall prevalence of cooperation remains approximately constant. In addition, there is a narrow range of conditions that exhibit chaotic diffusion patterns. As Nowak and May [1] demonstrated, the prevalence of cooperation soon orbits the time average in even these extreme conditions, and thus the average remains a representative description of the model’s central tendency. The model’s dynamic behavior in this tiny region is described by Nowak and May, and does not play a role in the experimental findings that we present. We run the simulation until it reaches equilibrium, a closed cycle of states, or a maximum number of rounds (replicated for both 500 and 10,000 iterations, with identical results). Given questions about sensitivity of the Nowak and May model to asynchronous updates of agents’ strategies [68, 69], we confirmed that our results are robust to this issue: First we replicate the original spatial dilemmas model on a Moore neighborhood with synchronous updates, and then we apply it to an alternative network topology (an empirical network of organizations) with random asynchronous updates.

We will show that the model’s behavior is highly sensitive to the initial configuration of cooperators (particularly whether there exists at least one sufficient cluster of cooperators at the random start), but even in these cases the average over many runs of long-run cooperation is a meaningful representation of the relative viability of cooperation.

Without loss of generality, we fix the Value of exchange at an arbitrary positive value (V = 8). We then manipulate the benefit of unilateral defection (‘Greed’ or G) and the cost of unilateral cooperation (‘Fear’ or F) over 50 evenly-spaced intervals in the broad range (0,8], encompassing the full range of the model’s behavior in the PD. Because the initial level of cooperation, C(0), may moderate the effects of Greed and Fear, we also manipulate this variable. At one extreme, we assume that only 10% of the population is already cooperating (90% is defecting) at the start of the simulation. At the other extreme, we assume that 90% of the population is already cooperating (10% is defecting). We also explore intermediate levels of 30%, 50%, and 70% initial cooperation. This manipulation of initial conditions demonstrates the robustness of our conclusions and provides diagnostic clues about the model’s behavior. Also, manipulating initial conditions helps translate insights from the model to inform interventions under empirical scenarios where cooperation may already be widespread or where defection may be rampant.

**Fig 1:**
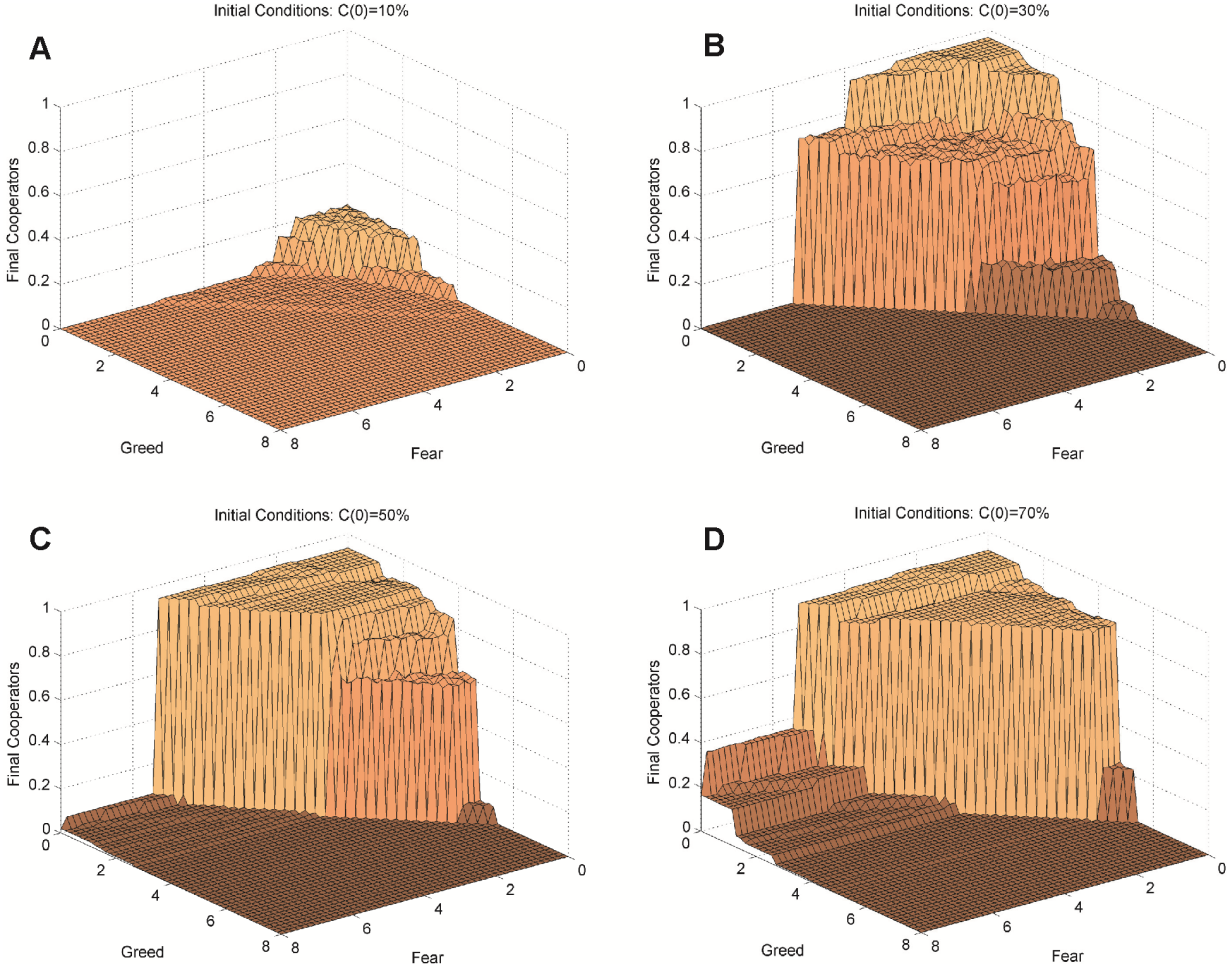
Long-run cooperation by Greed by Fear, with four levels of initial cooperation. (A) 10% Initial cooperators. (B) 30% Initial cooperators, (C) 50% Initial cooperators. (D) 70% Initial cooperators. The effects of Greed and Fear on long-run cooperation are highly nonlinear, with cooperation exploding or collapsing at particular combinations of Greed and Fear. With initial cooperators randomly dispersed, higher initial levels of cooperation generally make cooperation more viable at the system level, because the viability of cooperation depends on local clusters of cooperators. However, the relationship between initial cooperation and long-run cooperation is not simple and may even be non-monotonic in some conditions, depending on the levels of Greed and Fear.

**Fig 2:**
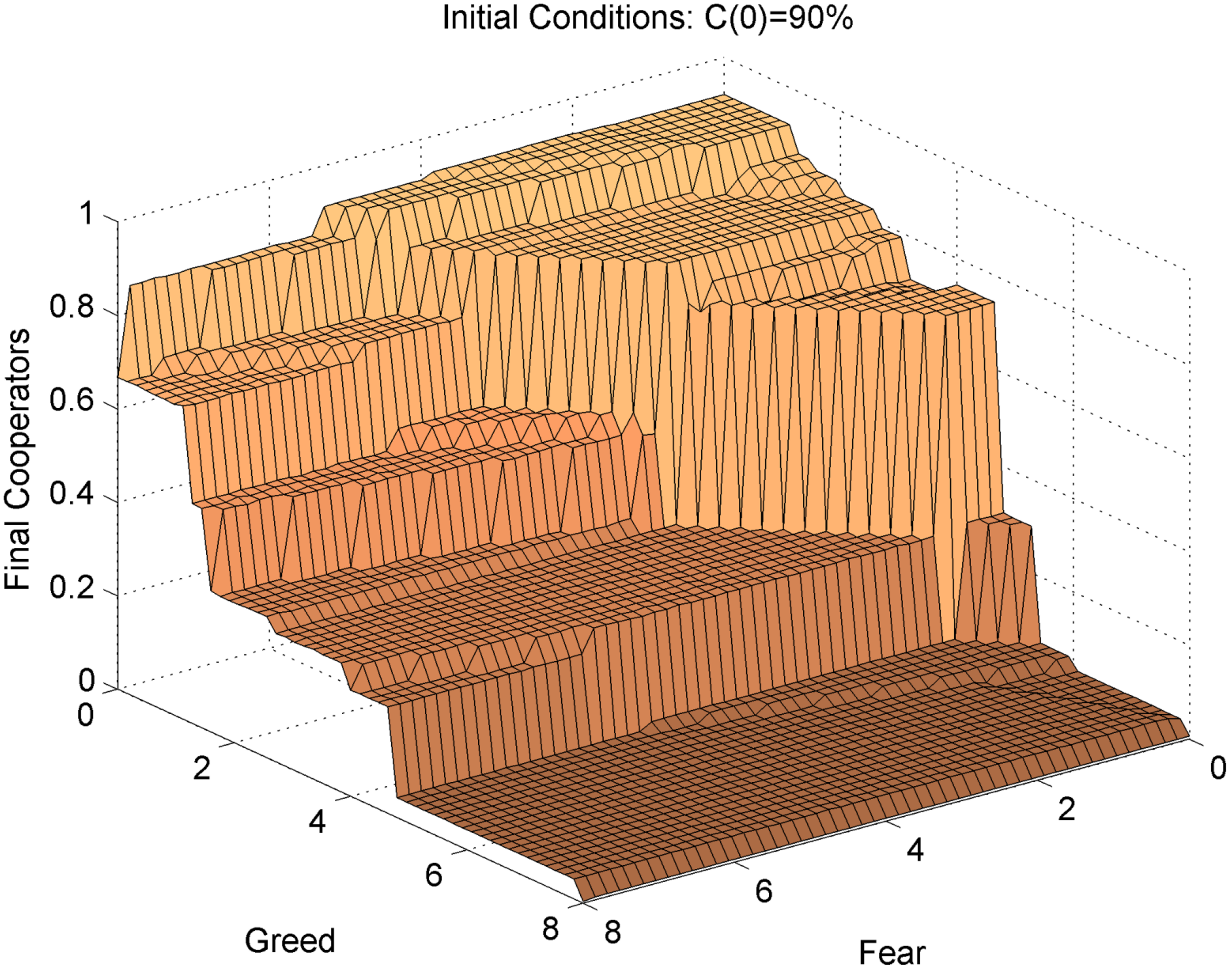
Long-run cooperation by Greed by Fear, with 90% initial cooperators. Results for high initial cooperation (90%) offer the most general and revealing view of the effect of Greed and Fear on cooperation. This highlights that the effects of Greed and Fear are nonlinear, as cooperation explodes (or collapses) at critical values of Greed and Fear. Although individual agents do not respond differently to Greed or Fear, the system-level effects of these two parameters of the dilemma are quite different, and these effects are interactive: The figure shows that there is a primary cliff that is a function of both Greed and Fear. When Fear is high, cooperation is a gradually decreasing function of Greed. When Fear is low, Greed has little effect on cooperation over the bottom half of the range, and then cooperation falls steeply. At both low and high levels of Greed cooperation is relatively insensitive to Fear, but at middle levels of Greed cooperation plummets as Fear exceeds a moderately low value.

Considering the manipulations of F, G, and C(0), we may regard the research design as a 51x51x5 factorial experiment. Because the outcome of each simulation run is affected by random factors and thus there is outcome variability within conditions, we replicate the simulation 1,000 times in each condition to derive a stable and precise estimate of the model’s mean behavior in that condition. The experiment then includes 2,601,000 independent observations (51 x 51 x 1,000) of the long-run simulation outcomes at each of the five initial levels of cooperation, allowing us to see the shape of the model’s average convergent behavior as these parameters are manipulated. Specifically, we plot *mean cooperation* across the entire range of Greed and Fear as a behavioral surface. We present five surfaces, one at each level of C(0) = 10%, 30%, 50%, 70%, and 90% in Figs 1 and 2 below. The fine-grained manipulation of independent variables and large number of replications give confidence that we accurately portray the average level of cooperation in the model at convergence. We eschew statistical analysis, which would allow us to reject the null hypothesis that the true surface is flat, in an infinite population of simulations under the model. Given our sample of 13,005,000 runs, our estimated behavioral surface is indistinguishable from the model’s true mean response, and it is obviously not flat.

## Results

Before presenting our experimental results, we describe the behavioral dynamics underlying the macro-level patterns. We invite readers to observe these dynamics directly by consulting animated video figures showing typical simulation runs at exemplary values of Greed (G), Fear (F), and initial cooperation C(0). Readers can also directly access the data from simulations and a platform-independent (NetLogo) program that illustrates the local dynamics and allows replication of the experiments in this paper (see this link).

### Qualitative Behavioral Dynamics

After the matrix is ‘seeded’ with cooperators and defectors randomly according to C(0), the population distribution changes over time, with strategies appearing to migrate across the network by diffusing among neighbors. In some regions of the payoff space (high Greed), defection marches across the population and cooperation is typically eliminated after only a few rounds. Even under these hostile conditions, however, cooperation is able to survive if sufficiently large clusters of cooperators appear with sufficiently clear boundaries (smooth edges that minimize contact between cooperators and defectors). Specifically, in the payoff region we consider, cooperation is always stable if a rectangular cluster of at least 3x3 cooperators forms and its edges are sufficiently clear, i.e. without interloping defectors inside the cluster or “satellite” cooperators near the cluster’s edge. In conditions of high Greed, survival of cooperation depends on the exogenous appearance of cooperator clusters, and those clusters can survive but cannot grow. See S1 Video for a sample simulation in this *stagnation* region of the payoff space (Fear = 8; Greed = 8).

In other regions of the payoff space (where values of Greed are lower), rough edges of cooperator clusters fill in with other cooperators and stable enclaves of cooperation form. Cooperator clusters are still necessary to get the process started, but in these payoff conditions the cooperating cluster is able to tolerate porosity at cluster edges and is able to strengthen the community by clarifying those edges over time, but still cannot diffuse broadly. See S2 video for a sample simulation in this *consolidation* region (Fear = 6; Greed = 4).

In regions of low Greed and Fear, clusters of cooperation of sufficient size and coherence can spread across the network, leaving scattered clusters of defectors surrounded by cooperators. Even here, however, the onset of cooperation depends decisively on the structure of interaction among cooperators. Sets of three or fewer cooperators always succumb to neighboring defectors who exploit them, regardless of the levels of Greed and Fear. By contrast, for very low Greed and Fear, smooth interconnected clusters (as small as four cooperators) perform better than neighboring defectors and so cooperation spreads. See S3 Video for a sample simulation in this *diffusion* region (Fear = 0.1; Greed = 0.1). The video begins with a low initial level of cooperation to illustrate how cooperation can survive only in sufficient clusters, even in this most conducive corner of the parameter space.

### Experimental Results: Mapping the Behavioral Surface

Having described the qualitative dynamics of the model, we now map the effects of Greed, Fear, and initial cooperation on long run average cooperation, represented as the height of the surface in Fig 1.

First look at Fig 1A (10% initial cooperators). The far corner is a region where Greed and Fear are low, whereas the near corner shows conditions where Greed and Fear are near the maximum values examined. The low surface over most of the parameter space reveals that cooperation disappears entirely in almost every run. The plateau in the back corner, where Greed and Fear are low, seems to suggest a moderate level of cooperation, but this is misleading. In fact, it represents an explosion of cooperation in a small number of simulation runs and extinction of cooperation in the remaining runs, resulting in a moderately low mean.

Next, note that as the *initial* prevalence of cooperation increases from 10% in Fig 1A to 30% in Fig 1B, mean cooperation in the long-run increases over a region of the parameter space. Cooperation increases in other regions between 30% and 50% initial cooperation (Fig 1C) and between 50% and 70% initial cooperation (Fig 1D). Analysis of model dynamics reveals that this positive relationship of initial cooperation to long-run cooperation occurs because it increases the probability of that clusters of cooperation of sufficient size and coherence will appear and support the emergence of further cooperation. Without such clusters, even prevalent initial cooperation will fail to survive; with such clusters, cooperation may survive (and in some cases thrive) from even a negligible start. In some regions this effect of initial level of cooperation—e.g., moving from C(0) = 30% to C(0) = 50%–is surprisingly *non-monotonic*. For example, if one compares the maximum heights in back corners of Fig 1B and 1C, the former is almost 10% higher than the latter. This surprising result shows that increasing initial cooperation can decrease average long run cooperation because, unlike cooperators, defectors are harmed by ties to others like themselves. Clusters of defectors can perform poorly and be overridden by local cooperator enclaves in this corner. Once C(0) is high enough that cooperator clusters form easily—given the levels of Greed and Fear—increasing C(0) further actually increases the proportion of defectors that are isolated, and thus able to ‘prey’ in purely cooperative neighborhoods. In fact, the scenario in which a single defector is located at the center of very large cooperative cluster has been described as the ‘defectors paradise.’ [11]

Fig 1B, 1C and 1D (30%, 50%, and 70% initial cooperators, respectively) highlight a stronger finding: Greed and Fear produce highly *nonlinear* effects on cooperation. Over portions of the parameter space—such as when Greed is relatively high—average cooperation hardly depends on either Greed or Fear. In other words, reducing Greed or Fear should have little marginal impact in this region. In contrast, when Fear is high and Greed is relatively low, long run cooperation is a decreasing step-function of Greed. Also, there is a large ‘cliff’ where decreasing Greed and/or Fear leads to a discontinuous leap in cooperation.

More surprising, although previous work on spatial dilemmas suggests that increased Greed and Fear have interchangeable negative effects on cooperation, our experiment reveals that the shapes of effects for Greed and Fear are different. This is not at all obvious, because we conventionally assume that individual agents respond identically to Greed and Fear. In order to cast the differential effects of Greed and Fear on mean cooperation in boldest relief, we focus in Fig 2 on interpreting the behavioral surface where cooperation among network neighbors is widespread at the start: C(0) = 90%. This allows us to focus on the divergent effects of Greed and Fear by reducing the distinct problem where cooperation fails due to the lack of a sufficient cluster at start. That is a real problem, but not the focus of our investigation in this section, so we proceed with initial conditions that include sufficient seed cooperators (avoiding the trivial extinction result that is largely independent of the parameters), and allow us to see the dependence of the model’s behavior on Greed and Fear.

Here we see clearly that the effects of Greed and Fear are nonlinear (cooperation explodes at critical values of Greed and Fear), and that Greed and Fear have divergent and mutually contingent population-level effects. In fact, the relationship between Greed or Fear and long run cooperation is surprisingly *non-monotonic* in some regions: Increasing Greed or Fear may lead to higher cooperation in some conditions. For example, if Greed and Fear are both near zero, increasing Fear to a moderate level (such as 3) marginally increases cooperation. If Fear is very low and Greed is moderate (around 4), then increasing Greed also marginally increases cooperation. These effects are robust to sampling error (not random “fluke” results) and all of the plateaus, ridges, and cliffs on this behavioral surface are intelligible with respect to the underlying mathematics of the model. These counterintuitive patterns also remind us that the model’s behavior is not obvious. However, here we briefly describe the overall shape of the behavioral surface, identifying features or patterns that are robust to the technical details of the model.

The online appendix gives a technical map of analytical boundaries in payoff space and a mathematical representation of why the model’s behavior changes at these boundaries. Each sharp ridge on the surface represents an analytical boundary where a particular configuration of cooperators (such as a four-party alliance, or a dense enclave with a protected interior) may become locally stable, or may spread by converting neighboring defectors. Greed and Fear have different effects because these various payoff boundaries may depend more or less on Greed or Fear. For example, large clusters of cooperators with high network closure (where some cooperators are tied to their neighbors’ neighbors) are relatively insensitive to Fear. This is because the stability of such cooperative enclaves rests on how well the neighboring defectors perform relative to the protected interior cooperators (reflecting Greed), not how much the cooperators on the cluster edge are hurt by being exploited (Fear). Once cooperators appear in enclaves, such that they have access to cooperative ‘role models’ who are relatively protected from exploitation, Fear has little effect on cluster stability.

Exceeding particular critical values of Greed (such as 1.14 or 4.8) in Fig 2 allows defectors to invade certain configurations of cooperators, leading to drops in cooperation. If these ridges are ‘horizontal’ in Fig 2 this means the effect is independent of Fear. By contrast, the primary ‘cliff’ in Fig 2 is diagonal because it depends equally on Greed and Fear. It is located on an analytical boundary in payoff space where a mass of cooperators adjacent to a mass of defectors will have equal performance along a smooth frontier separating the Cs from the Ds. If Greed and Fear fall jointly below this level, a cluster of cooperators can surpass a local ‘tipping point’ [70], sponsoring a cascade of cooperation. However, if Greed and Fear jointly exceed this critical level, no front line of cooperators can spread in this way, regardless of cluster size or shape, as neighboring defectors will resist any invasion.

Greed and Fear also have interactive effects—i.e. the effect of Greed on cooperation depends on the level of Fear. For example, compare in Fig 2 the effects of Greed on cooperation when Fear is high versus low. When Fear is relatively high, mean cooperation decreases in steps as Greed increases. Here cooperation spreads only by *consolidation* of local clusters. That is, initial clusters of cooperators ‘self-organize’ into cooperator enclaves, shoring up their boundaries and forming a coherent front against surrounding defectors. Access to protected interior colleagues bolsters them against the impact of exploitation by defectors, but they are not able to perform well enough for cooperation to diffuse further across the network.

In summary, we describe three distinct regions of the model’s qualitative behavior. When Greed and Fear are both relatively low, we see *diffusion* of cooperation across the network. When Greed is at least moderately low (but outside the *diffusion* region), we see *consolidation* of cooperator enclaves in a sea of defectors. In this region, cooperation can be stable and grow locally but it cannot diffuse broadly, so productive outcomes require some infusion of cooperation that is exogenous to the model (e.g., random initial clusters of cooperation). The level of cooperation at equilibrium here is a decreasing function of Greed (horizontal steps) and a decreasing function of Greed and Fear (diagonal steps). When Greed is high, we see *stagnation*: cooperation can survive only in large clusters that appear exogenously and such clusters cannot grow at all (as converting Ds to Cs is not possible), while variation of Fear in this region makes no difference because large clusters are unaffected by Fear. In all regions, the clustering of cooperators is crucial for the outcome observed. Importantly, our analytical investigation demonstrates that Fear plays a crucial role in whether cooperation can become established when cooperation is relatively rare and clustering of cooperators is weak.

### The Dynamics of ‘Virtual Interventions’ (Temporal Changes in Greed and Fear)

Readers should note that although Fig 2 is a static picture of long-run outcomes, the payoff boundaries previously discussed represent shifts in the *dynamics* of strategy diffusion, and these boundaries do not depend on the initial conditions or historical trajectory of the simulations. We show mathematically in the online appendix that the local stability of cooperation changes at these critical values, allowing us to discuss the dynamic implications of interventions in time. However, note that the surfaces in Figs 1 and 2 represent the average long run cooperation if the system begins at that point in payoff space. It does not necessarily follow that the system can ‘drive’ from one point in payoff space to another over time and ascend or descend the steps and hills on that surface. In fact, our examination of the dynamics of the model suggest that some of these analytical boundaries are *one-way*: The model’s dynamic behavior prior to the intervention affects the configuration of cooperators leading into the intervention, which clearly sets the initial conditions following the intervention.

We can intuitively begin with an illustration, provided in S4 Video: The first part of this video illustrates the typical evolution of cooperation (to an equilibrium of small enclaves) within the *consolidation* region (Fear = 2.0; Greed = 3.4). The second part of the video is an exogenous intervention that reduces Fear only marginally (from 2.0 to 1.9), but enough to cross into the *diffusion* region. In the simulation, the level of cooperation shifts from the inferior equilibrium to the cooperative equilibrium at the top of the cliff. This illustrates that even a tiny payoff intervention could transform dynamics as described, leading to widespread cooperation, as long as enclaves of cooperation survive the pre-intervention period.

In the *diffusion* region, over time cooperation can establish itself in large communities through spreading. The final outcome—widespread cooperation or extinction of cooperation—depends mostly on the presence of an initial cooperative enclave, and later changes to Greed and Fear do not make a large difference. If the intervention above is reversed to bring Fear from 1.9 to 2.0 (returning to the *consolidation* region), of course cooperation can no longer spread. However, cooperation does not collapse to its earlier sparse state. Instead, it remains at the elevated equilibrium. S5 Video shows that cooperation can be self-sustaining in large communities and typically persists at a high level after it has been bolstered for a sufficient duration. In substantive terms, if an intervention to reduce Fear lasts long enough to establish a coherent community of cooperation, then the community will be resistant to erosion of institutional support or other worsening of the social dilemma at a later time (and may even continue growing, to consolidate larger enclaves).

The simulation of interventions over time also suggests implications for interventions within the large *consolidation* region at the upper left portion of the payoff space. For example, we find that it is a general property of Fear interventions (more precisely, interventions that cross Fear-sensitive boundaries) that they are able to ascend steps on the behavioral surface in Fig 2, and any such changes tend to be internally reinforcing and thus difficult to reverse by later increases in Fear. Seen from the perspective of interventions, the steps on the Fear dimension tend to be one-way upward. Those are boundaries where cooperator clusters (of a particular size and coherence) are able to convert neighboring defectors, so dropping below them allows some defectors to convert and rising above them stops this process but does not reverse it. This is most striking for the primary ‘cliff’ at the boundary between the *consolidation* and *diffusion* regions.

If the interaction process operates for a sufficient time in the *consolidation* region, we know it will allow only isolated clusters of cooperation, which will be larger and more numerous if Greed is low (as we see in the horizontal steps of cooperation descending as Greed increases), when Fear is high. S6 Video shows that allowing Greed to climb higher (from 4 to 6) within the *consolidation* region will have dramatic effects, as the level of cooperation will easily descend the steps to a less cooperative equilibrium. However, in the less conducive conditions (higher Greed) the system loses the ability to ‘climb’ those steps even if an intervention diminishes Greed. S7 Video shows that again reducing Greed from 6 to 4 does not restore the earlier level of cooperation. The stairs in the *consolidation* region are generally harder to climb than to descend because increasing Greed in that region can destroy small clusters of cooperation, but decreasing Greed cannot resurrect clusters that no longer exist. In fact, where Fear is high (in the *consolidation* region), increasing Greed over time can have a substantial negative impact on cooperation, but decreasing Greed over time even the same amount may do little to improve cooperation. Whereas we saw that the diagonal steps on the Fear dimension may be one-way upward, the horizontal steps on the Greed dimension do not depend at all on Fear and tend to be oneway *downward*: That is, a slight increase in Greed that crosses one of those boundaries will erode cooperation, but then a slight decrease in Greed to return to the initial payoffs will not restore lost cooperation. The mathematical analysis in the online appendix explicates the dynamics of asymmetric effects of Greed and Fear interventions.

The asymmetry in long run behavior following temporal interventions provides further insight into the implications of Greed and Fear. If high Greed leads to tenuous distribution of cooperators (isolated in small enclaves), decreases in Greed may not be effective to jump-start cooperation within the *consolidation* region. For example, after beginning within the *stagnation* region at high Greed and high Fear, we may reduce Greed all the way to near zero with little effect on cooperation. The large horizontal steps on the Greed dimension represent critical values above which particular cooperator clusters become unstable, and are defeated by neighboring defectors (see online appendix). Thus, allowing Greed to exceed these boundaries kills cooperator clusters, and intervening to reduce Greed through these boundaries merely sets up a safe space for clusters that have already died.

When cooperation is confined to isolated enclaves, the most effective intervention is often to reduce *both* Greed and Fear to directly cross the threshold of the *diffusion* region and promote widespread cooperation. If Greed is in the moderate range, then interventions to Fear may also be effective in supporting the lasting spread of cooperation because the Fear-sensitive boundaries tend to be one-way upward. That is, cooperation increases due to falling Fear will be self-reinforcing and stable. This is a crucial part of a strategy to get cooperation established when it is currently sparse.

### Beyond the Moore Neighborhood: Application to Real-World Organizational Populations

Simplifying assumptions that made the model tractable and amenable to visualization and description could raise questions about the generalizability of our conclusions. In particular, our use of the eight-neighbor regular lattice (Moore neighborhood) obviously departs from the interaction networks observed in the empirical world, and even the network topologies considered in more complex evolutionary models. We employed the lattice with Moore neighborhoods because it is a simple topology that facilitates visualization and description of dynamics. Also, using this established topology facilitates comparison to decades of previous work. For many other network topologies, network clustering is entangled with network size and density, which complicates interpretation of experiments. But in Moore neighborhoods, clustering is invariant and thus independent of network size and density. The Moore neighborhood is thus desirable as a simple substrate for our study of Greed and Fear.

The Moore neighborhood is obviously stylized, with a high level of local closure, long path lengths, and homogeneous degree distribution (equal number of network neighbors). First we note that there is a widespread propensity toward network closure in a variety of organizational networks—which may be due to localization in geographic, sociopolitical, or market space, or because organizations choose to link with partners of their partners for increased trustworthiness [71, 72]. Indeed, the assumed level of network closure in Moore neighborhoods is comparable to many empirical networks. The network average clustering coefficient [73] for a Moore neighborhood is 0.43 –invariant to the size or density of the network—which is within the bounds of observed clustering values for many empirical inter-organizational networks [72].

Other features of the Moore neighborhood are obviously unrealistic, such as the homogeneity of agents' degree (i.e. number of network neighbors) with long paths connecting some agents in the network. Indeed, previous research (e.g., [24, 74–77]) has shown that features of a social network held constant here (such as density, centralization, or closure) may affect cooperation. Effects of network topology on cooperation—while complex and interesting—are orthogonal to our lens here, and our qualitative conclusions for the general effects of Greed and Fear are broadly robust to the network topology. In sensitivity analyses, we find that even though the level of cooperation may vary across topologies, the main qualitative conclusions of our experiment (i.e. that Greed and Fear have strong, nonlinear, and mutually divergent and interdependent effects on cooperation) are also supported with the network structures of a cycle, a triangular/tetragonal/hexagonal grid, regular random graph, random graph, or small-world network. We systematically examine the effect of network topology in companion research, so here it is sufficient to note that our main qualitative conclusions about the payoff space are not an artifact of the particular network topology we use for the experiments.

Following recent work on the evolution of cooperation using simulations on empirical social network data [59, 78, 79], we further show robustness by replicating our experiment using real world data on inter-organizational relationships as a social substrate instead of the stylized Moore neighborhood. We began with a list of 4,416 board members of 2,450 of the wealthiest US corporations as of 2011–12, drawn from Domhoff’s [80, 81] publicly available dataset on elite U.S. corporations and their boards of directors. We drew a link between any two corporations that share at least one board member. This produced a single giant component of 2,400 corporations (after excluding 50 corporations that were not connected to the others), representing 98% of the original set of corporations. This empirical network is characterized by small distances between pairs of organizations (mean path lengths 4.44) and relatively high levels of clustering (clustering coefficient 0.51), which are defining characteristics of small world networks [72, 82, 73]. At the same time, however, this network is also characterized by a heterogeneous and skewed degree distribution (mean degree = 7.43; standard deviation = 6.57; range = 1–47), which has been shown to be important in research on scale-free networks due to the role of hubs (highly connected agents) in the evolution of cooperation [21, 23, 82]. Aside from the short path lengths and degree heterogeneity of the organizational network, the organizational network is surprisingly very similar to the regular lattice with Moore neighborhoods. The size is 2,400, whereas the Moore neighborhood is 2,500. The mean degree of the organizational network (7.43) is only slightly below that for the regular lattice (8.0), the density of both networks is surprisingly identical at 0.003, and the clustering coefficient is 0.51 (vs. 0.43 for the Moore neighborhood).

We run the same simulation as for the Moore neighborhood using the empirical organizational relationship data as a network substrate. Again, we begin with 90% cooperation as an initial condition, with initial behavior assigned randomly. As a further step beyond the original spatial dilemmas model, here we update organizations’ states in random asynchronous fashion, but otherwise the update rule was the same as the one used for the regular grid.

**Fig 3:**
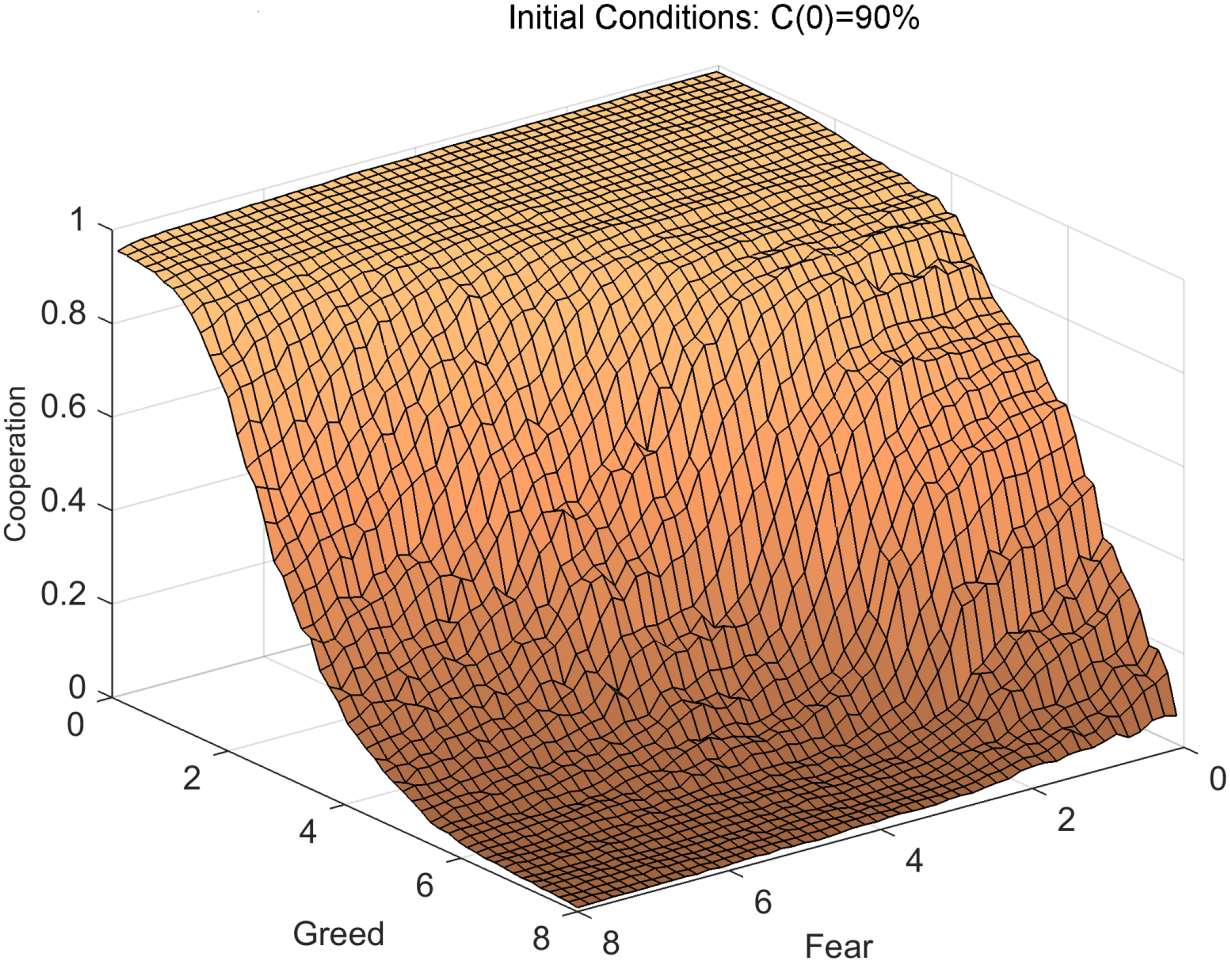
Replication of experiment on empirical inter-organizational network data, with 90% initial cooperators. The model is applied to empirical data on relationships among 2,400 corporations, which are similar in several ways to the Moore neighborhood (high clustering, similar network size and density and thus similar average neighborhood size) but also include short path lengths and a skewed degree distribution. The figure shows that there is a primary cliff that is a function of both Greed and Fear. When Fear is high, cooperation is a gradually decreasing function of Greed. When Fear is low, Greed has little effect on cooperation over the bottom half of its range, and then cooperation falls steeply. At both low and high levels of Greed cooperation is relatively insensitive to Fear, but at middle levels of Greed cooperation plummets as Fear exceeds a moderately low value.

Fig 3 shows that the model’s response to Greed and Fear in the context of the network of elite U.S. corporations looks qualitatively similar to the original surface with a regular grid: At high levels of Fear, cooperation falls rapidly with Greed and then flattens out at the middle range of Greed. At low levels of Fear, cooperation stays high until the middle range of Greed and then falls rapidly in the high range of Greed. There is also a primary cliff for the diffusion boundary, which depends equally on Greed and Fear. Finally, cooperation is relatively insensitive to Fear at high and low levels of Greed, but depends nonlinearly on Fear in the middle ranges of Greed. These simulations demonstrate that our qualitative interpretation of the surface in Fig 2 is robust to significant topological variations that go beyond the highly stylized regular lattice with Moore neighborhoods. The previous general claims for *diffusion*, *consolidation*, and *stagnation* regions still apply as guiding insights, but cannot be simply visualized and analyzed as they could for the Moore neighborhood. In fact, the relatively tractable set of boundaries that exist for regular grids multiply into a large number of boundaries because many more combinations of C and D neighbors become possible in a degree heterogeneous network. Indeed, the large number of configurations that can arise are one of the main reasons why games on complex (e.g. irregular) graphs are better studied through computer simulations rather than by purely analytical means. Thus, Fig 3 appears ‘smoothed’ but the qualitative shape from the experiment on Moore neighborhoods is retained.

Our investigation of the model’s behavior on a real-world organizational network serves two purposes. First, it demonstrates that the Moore neighborhood in the original spatial dilemmas model is surprisingly representative of a prominent inter-organizational network in some respects, such as network size, density, and clustering. By incorporating other features of real world organizational networks (such as short path length and skewed degree distribution) that are not present in the conventional spatial dilemmas model, we demonstrate the robustness of findings presented here for Greed and Fear to other features that appear in naturally occurring organizational networks.

### Robustness of the Core Results

Another generalizability concern for any simulation experiment is the sensitivity of results to initial conditions in the simulations. As described earlier, the patterns in the behavioral surface *do* reflect the initial conditions for the simulation, but this question is substantively meaningful; investigating the role of the initial prevalence of cooperation has given an important perspective on our experiment. We manipulated initial cooperation to explore a range of the model’s behavior, and thus inform a variety of different contexts, but we focus most interpretation on C(0) = 90% in Fig 2. Here we present four reasons that the Fig 2 is the most general and robust view: First, if we had allowed for exogenous shocks, such as cultural, political, or economic events that would generate a surge (or crash) of cooperation, such shocks would make the long-run behavior less dependent on initial conditions, and the analytical boundaries in payoff space would remain the same. Second, if we had allowed an exogenous tendency for cooperators to cluster together (by signaling, reputation, or some other mechanism), this mechanism would allow the dynamics we have seen to operate more easily at lower initial levels of cooperation, and it would not affect the boundaries in Fig 2. Third, if we had allowed for error (or exploration) in this model, then even where cooperation is absent at start, random trials of cooperation will eventually form clusters of sufficient size to support cooperation, although noise can also disrupt such clusters. Investigating noise is the focus of much other research [11, 13, 83]. Our focus here on payoff space and analytical boundaries for stability of different configurations of cooperators is equally relevant to models with noise and network dynamics, and we pursue those connections in other work because they are outside our scope here.

Computational experiments require extensive sensitivity analyses and robustness checks to verify the operation of the model and interpretation of the results. In this case, such checks have ensured that the core results are stable and robust to sampling error; they do not differ if we run simulations for longer runs, or a larger number of runs. The exact boundaries on the behavioral surface reflect the specification of our embedded dilemmas model, but the overall shape that we have interpreted in this paper remains robust as we relax numerous specific assumptions in the model. Because these various technical details do not alter the main conclusions that we have reported and interpreted here (about the strong, nonlinear, and mutually divergent and interdependent effects of Greed and Fear), we present the simpler established spatial dilemmas model. Finer details may depend on the specifics of the model, and some of these sensitivities are covered in more comprehensive technical studies of the spatial dilemmas model [8].

## Discussion: Implications for Organizations and Policy

Simulation research helps to fortify our intuitions in considering complex phenomena, guiding us as we move beyond the capabilities of simple analytical models and well-controlled empirical experiments. It allows us to isolate and investigate particular factors that may be difficult to separate in available empirical data. In this paper we have used this rigorous lens to investigate fundamental questions about inter-organizational cooperation using an established model of strategic interdependence and social learning. This section will offer some speculative thoughts about how the model may offer insights to concrete real world problems in organizational populations. Because the model is very stylized and abstract, we should of course exercise caution in applying these insights to the empirical world. Establishing the practical value of these insights will require sustained empirical inquiry and further theoretical elaboration.

### Economic Relations as Social Dilemmas

The success of many organizational strategies depends on the cooperation of other organizations [84]. While collectively beneficial, acts of cooperation involve relying on others, making firms vulnerable to betrayal. In the language of game theory, such *defections* include not only blatant acts of opportunism—e.g., appropriating knowledge generated through joint efforts or contributing fewer resources than promised—but also more subtle transgressions such as lapses in quality control or complacency about meeting partner needs. Such social dilemmas often arise in the context of inter-organizational interaction [85]. The value of voluntary cooperation in social dilemmas as a foundation for mutually beneficial economic exchange is widely accepted. Among other benefits, cooperation speeds up transactions, reduces transaction costs, promotes organizational learning, makes alliances more productive, and is a key element of task specialization and the division of labor [84, 86–88]. Identifying mechanisms to influence cooperation is thus a key area of concern for social scientists.

A classic way to ensure cooperation in social dilemmas is through the rule of law. Indeed, contract enforcement is widely acknowledged as a key ingredient to both inter-organizational cooperation and collective prosperity [89–91]. In political science, the canonical public interest model of regulation—endorsed by many who study political economy [92, 93]–emphasizes the ways in which cooperative, alliance-based strategies falter when the government fails to enforce contracts by sanctioning perpetrators and compensating victims [94]. And, as noted by Douglas North, ‘[T]he inability of societies to develop effective low-cost enforcement of contracts is the most important source of both historical stagnation and contemporary underdevelopment in the Third World’ ([95]: p. 54). The political economics literature has also provided support for this assertion, finding that governments that enforce contracts more rigorously and responsibly enhance national wealth and economic growth, often in a nonlinear exponential way [89, 96]. These results are compatible with a conclusion that companies within these nations can confidently cooperate with each other when the situation calls for interdependent value creating activities [90].

An important point made by North’s theory of institutions and economic performance is that contracts can be enforced by multiple parties—i.e., from governments [92, 93, 97], industry groups [98], or collectives of alliance partners [86]. As we focus on the logical consequences of interventions to Greed or Fear, our general argument is agnostic as to which body provides the regulation or other intervention. While sanctioning offenders is an assumed characteristic of governmental contract enforcement, research shows that sanctioning is also a key component of private regulation arrangements [99, 100, 101], including the use of rules, active monitoring, and punishment for infractions. But whether done through government or industry standards, the underlying mechanism is the same: A powerful third party develops institutional rules of fair exchange and then enforces those rules by punishing defection and/or compensating victims of defection.

If third party contract enforcement is essential to facilitating productive exchange and economic prosperity, the question arises: why are there not (even) more efforts by public or private actors to develop interventions that enforce contracts and otherwise promote ethical business practices? One possible answer is the belief that massive and costly efforts—i.e., determining rules, monitoring behavior, adjudicating disputes, and sanctioning transgressors—will yield little benefit because, in order to succeed, they must completely *solve* the dilemma by making cooperation more beneficial than defection in *any* given interaction. If it is in fact necessary to monitor and sanction defectors so severely that they no longer have a direct incentive to exploit cooperators, such a draconian system of monitoring and punishment would be a prohibitively expensive solution to the social dilemma. On the other hand, research on spatial dilemmas and network reciprocity suggests that interventions can foster cooperation on networks even without solving the social dilemma in dyads. The plausible assumptions of interdependence of agents’ choices and the proliferation of strategies by relative performance suggest that institutional changes that affect the relative payoffs for cooperation or defection can compound to rapid and accelerating trends. Understanding the positive feedback loop implied by this interdependence offers some traction for responding strategically to the trajectory of cooperative economic activity.

We have seen that these cascades can work both ways. Under some conditions, even marginal interventions to the payoffs for cooperation can lead to cooperation diffusing through interaction networks, establishing robust communities of mutually reinforcing cooperation. Under other conditions, even large interventions to reduce incentives to defect in social dilemmas should have no appreciable effect on cooperation. Under still other conditions, allowing Greed to creep even slightly over key threshold values can lead to unraveling of the structural foundation of cooperation. Understanding the conditions under which these scenarios play out can be valuable to both policy makers and corporate strategists.

### Greed and Fear in Regulating Inter-Organizational Relations

Despite the common assumption that Greed and Fear play similar roles in social dilemmas, much of this paper has emphasized how embedding social dilemmas in networks yields divergent effects of Greed and Fear on cooperation at the population level. This has proven true even if individuals respond identically to Greed and Fear in the model. The fact that Greed and Fear are mutually contingent and interactive means that intervening to reduce one should always take the other into account.

Here we consider a different reason to consider Greed and Fear separately. These two forces represent distinct substantive problems with distinct families of solutions for public policy makers, self-regulatory bodies, or other institutional entrepreneurs. That is, empirically intervening to diminish the benefits for exploiting other firms may be very different from intervening to diminish the harm of being exploited by other firms. For example, legislation and regulation intended to promote economic competition—e.g., the passage of the Sherman Antitrust Act and the establishment of the Federal Trade Commission—contain distinct elements that seem intended to reduce Greed and Fear separately. First, some facets of antitrust policy are intended to reduce harm (Fear) to firms by compensating those that have been victims of discriminatory, predatory, or collusive pricing. Mitigation of harm due to such practices can increase the willingness of firms to enter (or remain in) markets and increase (maintain) competition in those markets. Second, the prohibition of monopoly in product markets reduces the Greed factor, as the benefit of engaging in anticompetitive practices diminishes markedly if monopoly or near-monopoly is not a viable goal. The relative effectiveness of these two types of institutional interventions is relevant to a nation’s competition policy.

Considering both the experimental results and the dynamic analysis, we find that the most direct way to promote widespread cooperation is often joint reduction of Greed and Fear, moving toward the corner where conditions are conducive for the diffusion of cooperation. Indeed, policy interventions may also aim to reduce Greed and Fear simultaneously. Restitution policies that would force a unilateral defector to compensate a victim for damages are an intuitive approach to accomplish this end. A typical manifestation of this approach is to make restitution easier through constructive changes in the law involving civil procedures. An added advantage of this approach is that it involves only interactions between parties involved in private contracting, rather than increased governmental regulation, an approach thought to be undesirable by some observers of politics.

Two non-governmental examples are also illustrative. Hagen and Choe [102] show that Japan’s use of trust to support interfirm cooperation addresses the reduction of both Greed and Fear, but through separate mechanisms. Elaborate *subcontracting practices*, including bilateral decision making processes and systematized evaluation procedures, work to substantially reduce the harm of being exploited by partners (Fear). *Social sanctioning*, including wide dissemination of reputational information, diminishes the advantages of cheating (Greed). Potential defectors may be deterred by both the difficulty of cheating and the difficulty of achieving any real gains from their misdeeds. Fear reduction is also a key element in ISO structures such as the ISO 9000 and 9001 standards that assure that high quality standards are met. ISO standards, however, are unlikely to have much effect on Greed. Thus non-governmental structures, like governmental mechanisms, work in various ways, but all aim to reduce either Greed or Fear, or both simultaneously.

If our investigating of Greed and Fear boundaries may inform tactics or general approaches, our investigation of initial conditions could inform strategic timing of intervention. We have seen that Fear is most relevant when cooperation is scattered, clusters are small, and enclaves are undeveloped. Regulators could take a lesson from our experiment to front-load interventions and establish momentum (especially with respect to Fear reduction) and then take advantage of the self-sustaining nature of cooperative communities to maintain a prosocial equilibrium even if the intervention itself is not sustainable. When the conditions for diffusion are not met, such as where institutional support is weak, cooperation can still survive in enclaves.

When cooperation is limited to established enclaves, however, it may be sensitive to Greed increases by erosion of institutional support, such as weakening enforcement of contracts or protection of intellectual property. Our analysis of virtual interventions shows that incremental decline of cooperation due to weakened institutional support (especially Greed increases) can be difficult to remedy in this region, as reversing increases in Greed cannot resurrect lost cooperative alliances. Under the worst conditions, managers might be well advised to abandon corporate operations dependent on inter-organizational cooperation, or move operations to more conducive environments. Regulators could recognize the importance of Fear interventions in these conditions to stabilize cooperation in small alliances. Fear grows less important as cooperation becomes established, when penalizing unilateral defectors may be a sufficient and simpler response. It makes sense to allocate early interventions toward reducing Fear (or Fear and Greed) until cooperative enclaves grow and become more structurally coherent, then simply avoid letting Greed increase.

For practical purposes, actors often have no way to manipulate Greed and Fear in their environment, nor even a clear and direct way to observe the value of Greed and Fear. It may still be possible to derive some lessons from this research. Where organizational collaboration is not strongly established, managers may be attentive to leading indicators of reduced institutional support as indirect signals to foretell an unraveling of trust. For example, indicators might include: 1) deterioration reflected in national corruption indices, regardless of current levels; 2) an increased incidence of scandals in economic relationships; 3) loosening of contract enforcement regimes, including proposed deregulation legislation, reduced funding for regulatory agencies, and decreased oversight by regulatory bodies; and 4) attempts to place individuals in enforcement roles based on political loyalty rather than competence and professional qualifications. Of course, our investigation tells nothing about the empirical link between such particular indicators and prospects for successful navigation of social dilemmas in economic exchange. What we may be able to address with insights from the model is temporal patterns in the evolution of cooperation as a result of those institutional changes. For example, if cooperation is not highly developed in substantial enclaves, and if leading indicators may be interpreted as increasing Greed (increasing rewards or decreasing penalties for exploiting others), then the model suggests that the context for cooperation may soon deteriorate in an accelerating fashion that is difficult to reverse or recover. Conversely, if leading indicators may be interpreted as decreasing Fear (increasing restitution or protections by government or other third parties), then the model suggests that the context for cooperation may grow in an accelerating fashion until substantial enclaves of cooperation develop.

### Clustering of cooperators

Our last substantive conclusion involves the structure of interaction among cooperators and defectors, which is a crucial ingredient of the processes we study but was not directly investigated in our experiments because we assigned strategies randomly to positions on the grid. We have shown that the stability of cooperation depends crucially on the organization of cooperators into clusters with high network closure, consistent with growing evidence that the structure of interaction is of paramount importance for organizational performance. The strategic context induced by institutional interventions (reduced Greed and Fear) may facilitate the diffusion of cooperation, but closure is necessary for survival of cooperators. Although we assumed initial random matching among agents (a standard assumption that gives an exemplary test case) one way to facilitate cooperation in practice would be to provide institutional support for matching partners that are predisposed to cooperate with one another, giving an advantage to cooperators in a footrace where predatory competitors also wish to partner with cooperators. Such interventions would certainly provide leverage for establishing cooperation when it is sparse and disorganized, conditions where even strong reductions in Greed or Fear may be insufficient. In addition, industrial groups with structures similar to Japanese Keiretsus [103] could also spawn local pockets of self-sustaining cooperation. Indeed, studies have shown that some geographic clusters of inter-organizational relationships in the biotech industry have developed cooperative norms [104, 105].

Finally, with caution given the level of abstraction of the model, managers may also take a couple of direct lessons from our findings about the network foundations of cooperation. When they have discretion for choice of strategic partners, managers may focus more due diligence on prospective partners’ interaction histories, rather than past performance, capabilities, and other assets. This due diligence can include such easily observed indicators as the durability of their past alliances and the tendency of their alliances to be embedded in networks with greater closure, which constrains defection. Of course, diligence is most crucial when the institutional climate and norms of cooperation are weak.

## Conclusion

Extensive research has exampled the ways that cooperation may evolved in spatially-organized agents in social dilemma situations. These literatures have examined many extensions (including alternative network topologies, different update rules, or behavioral noise) but overwhelmingly reduce the PD to a single dimension of severity. By investigating the distinct roles of Greed, Fear, and the initial level of cooperation in supporting sustained cooperation, we provide principled lenses for considering the environment for strategic interdependence in social dilemmas. To develop our understanding of the dynamics of cooperation in inter-organizational networks, we build on an established interdisciplinary research program in ‘spatial dilemmas.’ Specifically, we explore the robustness of cooperation when agents interact with network neighbors, and adjust their strategies through social learning. We show that this embeddedness of interaction generates macro-level patterns that are not at all obvious from the assumed micro-level dynamics.

First, although classical game theory suggests that variation of Greed and Fear (within the payoff boundaries of the PD) would have *no* effect on the level of cooperation, we show conditions where even a small difference in Greed or Fear may substantially influence the evolution of cooperation embedded in networks. Second, previous work on spatial dilemmas has assumed or argued that Greed and Fear are interchangeable as incentives to defect in the PD, and thus the severity of the game may be represented as a *single* parameter (i.e. the temptation to defect, the ratio of benefit to cost of cooperation, or the K-index of cooperation). We show that when social dilemmas are situated in networks, Greed and Fear may have divergent effects on cooperation at the macro-level *without* assuming that individual agents respond differently to Greed and Fear. Due to these highly nonlinear and interactive effects, and because Greed and Fear represent substantively different problems in organizational contexts, further research on inter-organizational cooperation should consider these two distinct motives for defection in social dilemmas.

Our experiments allow us to explore the shape of the expected level of cooperation under a range of conditions, providing a detailed map that illuminates the complex interaction of Greed and Fear. While the model is basic and abstract, the insights explicated here should be investigated in empirical research. Our virtual interventions take us one step beyond the experiment to explore some deeper insights about the dynamic consequences of changing Greed and Fear. For example, our analysis of virtual interventions in the *consolidation* region shows that boundaries in payoff space that are sensitive to Fear tend to be one-way upward in the sense that payoff interventions that cross these boundaries may lead to stronger and more coherent clusters of cooperation that can sustain even after the interventions cease. The superior performance resulting from cooperative communities becomes an emergent force for cooperation that makes those enclaves robust to later increases in Fear. By contrast, payoff boundaries that depend only on Greed tend to be one-way downward: They typically have negative effects on cooperation if crossed by an increase in Greed, but later interventions to decrease Greed cannot restore lost cooperation. In the *diffusion* region where Greed and Fear are both low, both widespread cooperation and extinction of cooperation are possible, depending mostly on the clustering of cooperators in the initial conditions. Later interventions are unlikely to make much difference within this region.

## Supporting Information

S1 Video Simulation in the *stagnation* region (Greed = 8.0, Fear = 8.0), with 90% initial cooperators. In the *stagnation* region with very high Greed and Fear, defectors (red) rapidly propagate over the lattice, until a single cooperator cluster (blue) is left at equilibrium. In this region, cooperation can survive only in rectangular clusters of at least 3x3 cooperators, without interloping defectors inside the cluster or “satellite” cooperators near the cluster’s edge. Such clusters must appear exogenously, and cannot grow.
(AVI)

S2 Video Simulation in the *consolidation* region (Greed = 4.0, Fear = 6.0), with 90% initial cooperators. In the *consolidation* region, rough edges of cooperator clusters (blue) fill in with other cooperators and stable enclaves of cooperation form in a field of defectors (red). Cooperator clusters are still necessary to get the process started, but in these payoff conditions the cooperating cluster is able to tolerate unclear cluster edges and strengthen the community by clarifying those edges over time, but still cannot diffuse broadly.
(AVI)

S3 Video Simulation in the *diffusion* region (Greed = 0.1, Fear = 0.1), with 30% initial cooperators. In the *diffusion* region with minimal Greed and Fear, scattered cooperators (blue) still switch to defection (red), but compact clusters of at least 4 cooperators not only survive but can spread into fields of defectors. The video begins with a low initial level of cooperation to illustrate how cooperation can survive only in sufficient clusters, even in this most conducive corner of the parameter space.
(AVI)

S4 Video Fear intervention, crossing from the *consolidation* region (Greed = 3.4; Fear = 2.0) to the *diffusion* region (Greed = 3.4; Fear = 1.9), with 90% initial cooperators. The first part of this video illustrates a typical evolution of cooperation within the *consolidation* region. After the model reaches equilibrium with a number of cooperator (blue) enclaves surrounded by defectors (red), an exogenous intervention reduces Fear from 2.0 to 1.9, crossing into the *diffusion* region. Cooperation then spreads rapidly from the enclaves, and the system climbs the ‘cliff’ to a much more cooperative equilibrium.
(AVI)

S5 Video Fear intervention, crossing from the *diffusion* region (Greed = 3.4; Fear = 1.9) to the *consolidation* region (Greed = 3.4; Fear = 2.0), with 90% initial cooperators. The first part of this video illustrates a typical evolution of cooperation within the *diffusion* region, with parameters identical to the second half of S4 Video. Cooperation rapidly spreads over the lattice. After the model reaches equilibrium with scattered defectors (red) surrounded by cooperators (blue), an exogenous intervention increases Fear from 1.9 to 2.0, returning to the *consolidation* region at the start of S4 Video. Although payoffs thus cross the same boundary in reverse, the system-level cooperation does not fall to the bottom of the cliff, but maintains the high level of cooperation. This illustrates the asymmetry in Fear interventions, which makes many Fear-sensitive boundaries one-way upward.
(AVI)

S6 Video Greed intervention within the *consolidation* region, rising from Greed = 4.0 to Greed = 6.0, with Fear = 4.0 and 90% initial cooperators. The first part of this video illustrates a typical evolution of cooperation within the low-Greed end of the *consolidation* region. After the model reaches equilibrium with cooperators (blue) settled into many large stable enclaves surrounded by defectors (red), an exogenous intervention increases Greed from 4.0 to 6.0. Unlike the upward Fear change in S5 Video, allowing Greed to increase leads the system-level cooperation to descend the ‘steps’ in Fig 2, resulting in a much less cooperative equilibrium.
(AVI)

S7 Video Greed intervention within the *consolidation* region, falling from Greed = 6.0 to Greed = 4.0, with Fear = 4.0 and 90% initial cooperators. The first part of this video illustrates a typical evolution of cooperation within the high-Greed end of the *consolidation* region, with parameters identical to the second half of S6 Video. After the model reaches equilibrium with cooperators (blue) settled into a few stable enclaves surrounded by defectors (red), an exogenous intervention decreases Greed from 6.0 to 4.0, returning to the same Greed value as the start of S6 Video. Although payoffs cross the same boundary in reverse, the system-level cooperation does not rise to the top of the step, but maintains the low level of cooperation. This illustrates the asymmetry in Greed interventions, which makes Greed boundaries one-way downward in the high range of Greed. Here (e.g. the boundaries at Greed = 8.0 and Greed = 4.8), allowing Greed to cross above the boundary destroys cooperator clusters and moving back below the boundary cannot revive those clusters.
(AVI)
